# Identification of Autoantibodies against TRPM1 in Patients with Paraneoplastic Retinopathy Associated with ON Bipolar Cell Dysfunction

**DOI:** 10.1371/journal.pone.0019911

**Published:** 2011-05-17

**Authors:** Mineo Kondo, Rikako Sanuki, Shinji Ueno, Yuji Nishizawa, Naozumi Hashimoto, Hiroshi Ohguro, Shuichi Yamamoto, Shigeki Machida, Hiroko Terasaki, Grazyna Adamus, Takahisa Furukawa

**Affiliations:** 1 Department of Ophthalmology, Nagoya University Graduate School of Medicine, Nagoya, Aichi, Japan; 2 Department of Developmental Biology, Osaka Bioscience Institute, Suita, Osaka, Japan; 3 JST, CREST, Suita, Osaka, Japan; 4 Department of Biomedical Sciences, Chubu University, Kasugai, Aichi, Japan; 5 Department of Respiratory Medicine, Nagoya University Graduate School of Medicine, Nagoya, Aichi, Japan; 6 Department of Ophthalmology, Sapporo Medical University School of Medicine, Sapporo, Hokkaido, Japan; 7 Department of Ophthalmology and Visual Science, Chiba University Graduate School of Medicine, Chiba, Chiba, Japan; 8 Department of Ophthalmology, Iwate Medical University School of Medicine, Morioka, Iwate, Japan; 9 Department of Ophthalmology, Oregon Health and Science University, Portland, Oregon, United States of America; Dalhousie University, Canada

## Abstract

**Background:**

Paraneoplastic retinopathy (PR), including cancer-associated retinopathy (CAR) and melanoma-associated retinopathy (MAR), is a progressive retinal disease caused by antibodies generated against neoplasms not associated with the eye. While several autoantibodies against retinal antigens have been identified, there has been no known autoantibody reacting specifically against bipolar cell antigens in the sera of patients with PR. We previously reported that the transient receptor potential cation channel, subfamily M, member 1 (TRPM1) is specifically expressed in retinal ON bipolar cells and functions as a component of ON bipolar cell transduction channels. In addition, this and other groups have reported that human TRPM1 mutations are associated with the complete form of congenital stationary night blindness. The purpose of the current study is to investigate whether there are autoantibodies against TRPM1 in the sera of PR patients exhibiting ON bipolar cell dysfunction.

**Methodology/Principal Findings:**

We performed Western blot analysis to identify an autoantibody against TRPM1 in the serum of a patient with lung CAR. The electroretinograms of this patient showed a severely reduced ON response with normal OFF response, indicating that the defect is in the signal transmission between photoreceptors and ON bipolar cells. We also investigated the sera of 26 patients with MAR for autoantibodies against TRPM1 because MAR patients are known to exhibit retinal ON bipolar cell dysfunction. Two of the patients were found to have autoantibodies against TRPM1 in their sera.

**Conclusion/Significance:**

Our study reveals TRPM1 to be one of the autoantigens targeted by autoantibodies in at least some patients with CAR or MAR associated with retinal ON bipolar cell dysfunction.

## Introduction

Paraneoplastic retinopathy (PR) is a progressive retinal disorder caused by an autoimmune mechanism and is associated with the presence of anti-retinal antibodies in the serum generated against neoplasms not associated with the eye [1]–[4]. The retinopathy can develop either before or after the diagnosis of a neoplasm. Patients with PR can have night blindness, photopsia, ring scotoma, attenuated retinal arteriole, and abnormal electroretinograms (ERGs). The diagnosis of PR is usually made by the identification of neoplasms and anti-retinal autoantibodies in the sera.

PR includes two subgroups: cancer-associated retinopathy (CAR) [5], [6] and melanoma-associated retinopathy (MAR) [7]–[10]. Although CAR and MAR share similar clinical symptoms, the ERG findings are very different. Both a- and b-waves are severely attenuated in CAR, indicating extensive photoreceptor dysfunction, whereas only the b-wave is severely reduced while the a-wave is normal in MAR, suggesting bipolar cell dysfunction [8], [9]. However, it was recently reported that cancers other than melanoma can cause bipolar cell dysfunction [11], [12]. Several autoantibodies against retinal antigens have been identified, but a specific antigen associated with bipolar cells has not been identified in patients with CAR and MAR [1]–[10].

In the current study, we identified autoantibodies against the transient receptor potential cation channel, subfamily M, member 1 (TRPM1) [13]–[15] in the serum of one patient with lung cancer. The ERG findings in this patient indicated a selective ON-bipolar cell dysfunction. We also investigated the sera of 26 MAR patients and found that two contained autoantibodies against TRPM1. Our results suggest that TRPM1 is one of the retinal autoantigens in at least some patients with CAR or MAR and may cause retinal ON bipolar cell dysfunction.

## Results

### Case report of CAR associated with ON bipolar cell dysfunction

**Figure 1:**
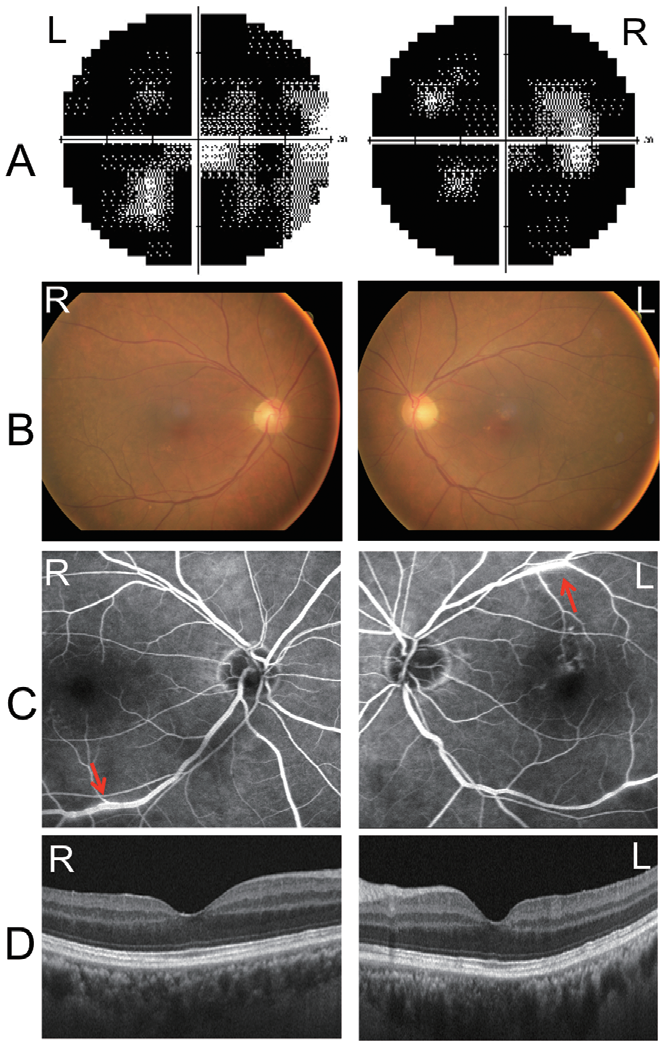
Ophthalmological findings from a patient with paraneoplastic retinopathy (PR) associated with lung cancer. (A) Threshold of static visual field (Humphrey, 30-2 program) plotted on a gray scale showing severely decreased sensitivities within the central 30 degrees of the visual field. (B) Fundus photographs of the patient showing a nearly normal fundus. (C) Fluorescein angiograms showing periphlebitis of the retinal vessels (arrows). (D) Spectral-domain optical coherence tomographic (SD-OCT) image of a 9 mm horizontal scan of the retina of our patient. The retinal structure in each retinal layer is normal.

A 69-year-old man visited the Nagoya University Hospital with complaints of blurred vision, photopsia and night blindness in both eyes of three months duration. At this point he was not diagnosed as suffering from any eye disease or systemic disease, including a malignant tumor, and his family history revealed no other members suffering from any eye diseases. On initial examination, his best-corrected visual acuity was 0.9 in the right eye and 0.6 in the left eye. Humphrey static perimetry revealed a severe decrease in sensitivity within the central 30 degrees of the visual field in both eyes (Fig. 1A). Dark-adaptometry of this patient showed a loss of the rod branch. The cone threshold was within normal range. Ophthalmoscopy showed a nearly normal fundus appearance except for slight hypopigmentation at the macula of the left eye, which may be due to age-related changes in the retinal pigment epithelium (Fig. 1B), but fluorescein angiography demonstrated periphlebitis of the retinal vessels (arrows, Fig. 1C). Spectral-domain optical coherence tomography (SD-OCT) showed that the morphology of the retina was normal in both eyes (Fig. 1D).

### Electrophysiological examinations

**Figure 2:**
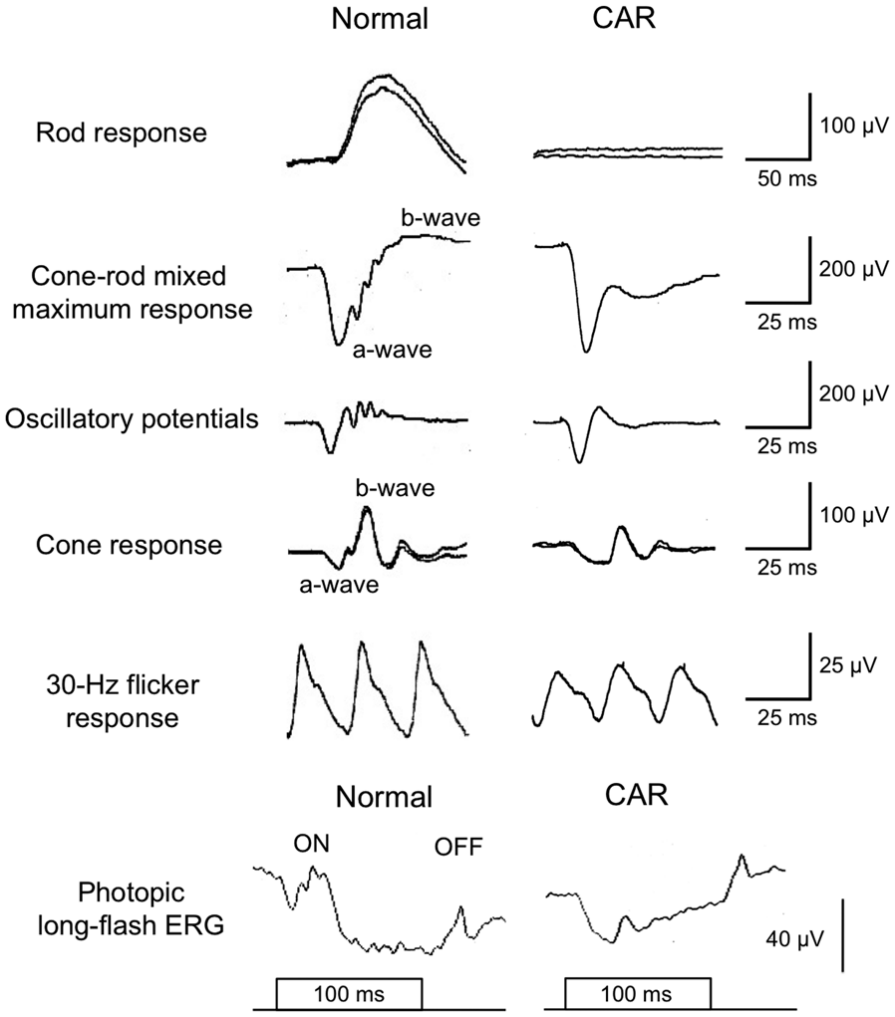
Full-field ERG recordings. The rod response was recorded with a blue light at an intensity of 5.2×10^−3^ cd-s/m^2^ after 30 minutes of dark-adaptation. The cone-rod mixed maximum response was elicited by a white flash at an intensity of 44.2 cd-s/m^2^. The oscillatory potentials were recorded with a white flash at an intensity of 44.2 cd-s/m^2^ using a band-pass filter of 50–1000 Hz. The cone response and a 30 Hz flicker response were elicited by a white stimulus of 4 cd-s/m^2^ and 0.9 cd-s/m^2^, respectively, on a blue background of 30 cd/m^2^. Photopic long-flash ERG responses were also elicited by long-duration flashes of 100 ms using a densely-packed array of white LEDs of 200 cd/m^2^ on a white background of 30 cd/m^2^.

Recordings of the full-field ERGs from this patient showed that the rod responses were undetectable (Fig. 2). The rod- and cone-mixed maximal response was a negative-type with an a-wave of normal amplitude and a b-wave that was smaller than the a-wave. The a-wave of the cone response had a wide trough, and the b-wave was reduced by 40%. The amplitude of the 30-Hz flicker ERG was reduced by 50%. The photopic long-flash ERG showed severely reduced ON response and normal OFF response. These ERG findings indicated that there was a defect in the signal transmission from photoreceptors to ON bipolar cells both in both rod and cone pathways.

Based on these ophthalmological and electrophysiological tests, we suspected that this patient might have PR and referred him to an internist. The general physical examination including positron emission tomography and computed tomography revealed two abnormal masses in the right lung. Biopsy of these masses confirmed that the masses were small cell carcinomas of the lung.

### Detection of autoantibodies against TRPM1 in the serum of the CAR patient

**Figure 3:**
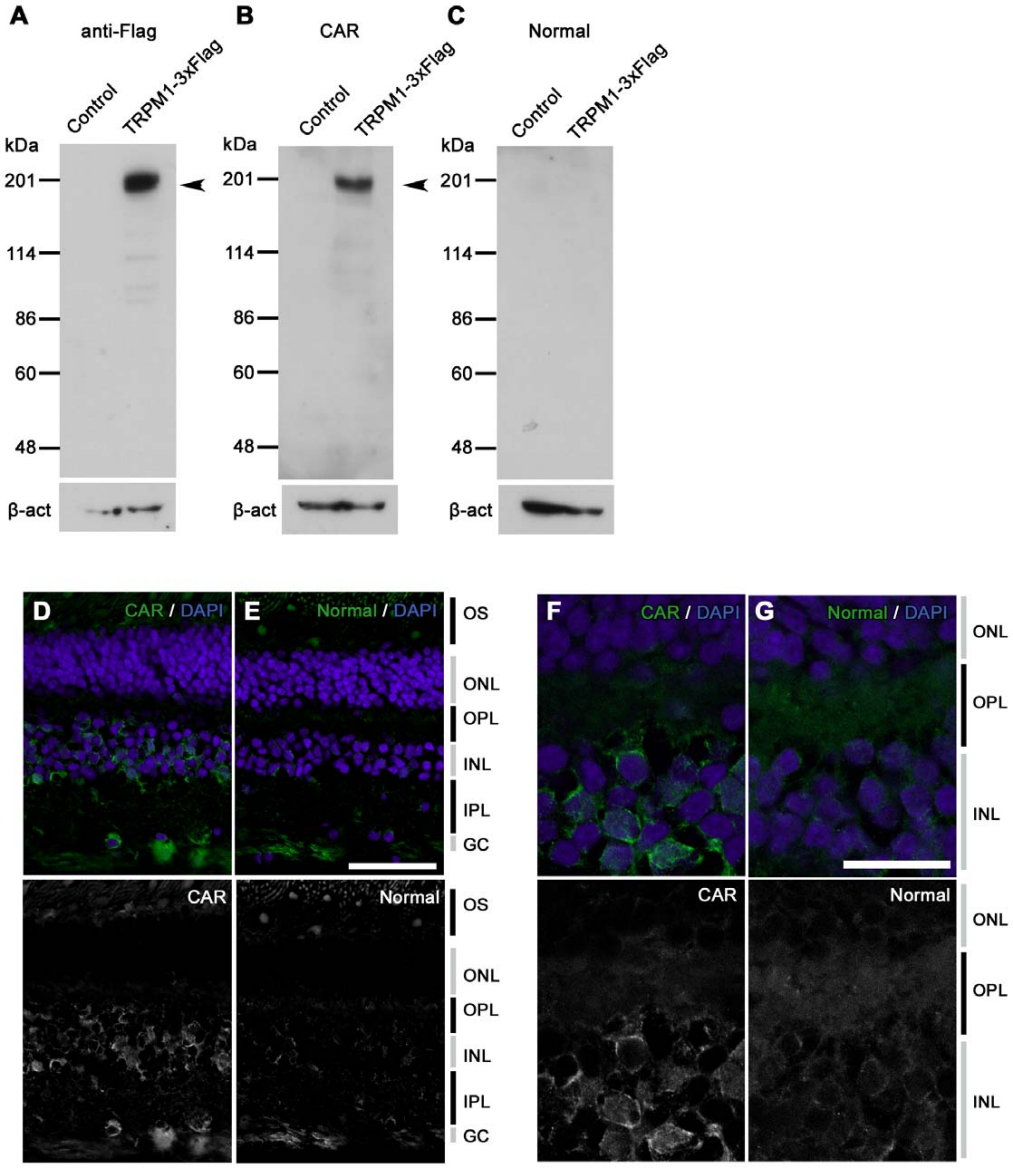
Immunostaining and Western blot analysis of human TRPM1 using serum from the CAR patient. (A–C) Immunoblots of the transfected cell lysates using an antibody against Flag tag (A), serum from CAR patient (B), and control serum (C). Arrowheads indicate the TRPM1-3xFlag protein bands. HEK293T cells were transfected with the pCAGGS or pCAGGS-human TRPM1-3xFlag plasmid, and cells were harvested after 48 hrs. β-actin (β-act) was used for a loading control. (D–G) Confocal images of a three-year-old rhesus monkey retina immunostained with the concentrated serum from the CAR patient (D, F) or the concentrated normal serum (E, G). Cell nuclei are visualized with DAPI. CAR patient serum presented signals on INL cells and the inner part of the OPL (D, F). Scale bar  = 50 µm in (E) and 20 µm in (G).

Based on our ERG examination results, we hypothesized that the serum of this CAR patient may contain autoantibodies against TRPM1. To test this hypothesis, we examined whether or not this CAR patient's serum could recognize human TRPM1 protein by Western blot analysis. We transfected an expression plasmid containing human TRPM1 cDNA with the C-terminal 3xFlag-tag (TRPM1-3xFlag) into HEK293T cells, and carried out a Western blot analysis using whole cell extracts harvested after 48 hrs cell growth. We first confirmed that TRPM1-3xFlag was expressed by cell using Western blot analysis and an anti-Flag antibody. We detected the ∼200 kDa TRPM1-3xFlag band in the cell lysates (Fig. 3A).

Next, we performed Western blot analysis on the same lysates using the serum from our CAR patient and a healthy control person. We detected immunostaining of the same size protein, which was confirmed with the anti-Flag antibody, and with CAR serum. The control serum did not present a significant band (Fig. 3B, C). This result showed the presence of autoantibodies against TRPM1 in this CAR patient's serum.

To examine whether the serum from the CAR patient recognized retinal bipolar cells, we carried out an immunohistochemical analysis on monkey and mouse retinas. We first performed immunohistochemistry on the retina of a 3-year-old rhesus monkey (*Macaca mulata*) and on the retina of a one-month-old C57/B6 mouse using the serum of the CAR patient, however, we did not obtain a significant staining signal above background (data not shown). We then concentrated the serum by IgG purification followed by filter spin column centrifugation and performed immunohistochemistry on the monkey retina using the concentrated serum (Fig. 3D–G). We observed a significant immunolabeling on the INL in the monkey retina (Fig. 3D, F) whereas the normal serum did not give a significant labeling (Fig. 3E, G). The antibodies immuolabeled both the bipolar side and amacrine side of the INL. Since most of the cells residing on the outer side of the INL are ON bipolar cells, at least some of the stained cells are ON bipolar cells. It should be noted some of the staining signals show a spotted pattern in the outer plexiform layer (Fig. 3F) as is observed in TRPM1 or mGluR6 immunostaining on the mouse retina [13], suggesting that the CAR patient serum recognizes the bipolar dendritic tips where some of the TRPM1 protein localizes.

### Western blot analysis of the sera from MAR patients

**Figure 4:**
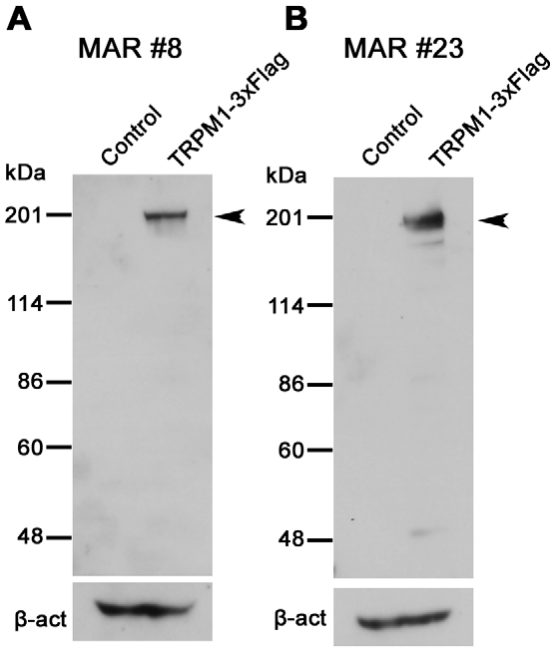
Western blot analysis of human TRPM1 using sera from the MAR patients. (A, B) Immunoblots of the transfected cell lysates using sera from MAR patient #8 (A) and MAR patient #23 (B). HEK293T cells were transfected with pCAGGS or pCAGGS-human TRPM1-3xFlag plasmid, and cells were harvested after 48 hrs. Arrowheads indicate the TRPM1-3xFlag protein bands. β-actin (β-act) was used for a loading control.

Since the functional defect in the retina of MAR patients is known to be due to abnormal signal transmission between photoreceptors and ON bipolar cells [8], [9], we then investigated whether or not autoantibodies to TRPM1 were also present in the sera of MAR patients. We obtained the sera of 26 MAR patients from two hospitals in Japan (Chiba University Hospital and Iwate Medical University Hospital) and Ocular Immunology Laboratory in the USA (Casey Eye Institute). We found that the sera from patients #8 and #23 exhibited a significant immunoreactive band against TRPM1-transfected cell lysates by Western blot analysis (Fig. 4A and B). The control serum showed no significant immune response against the TRPM1-transfected cell lysates (Fig. 3C). These results suggest that the sera from some MAR patients contain autoantibodies against TRPM1. Due to the limited volume of sera from the MAR patients, we could not try immunostaining on the monkey or the mouse retina using the serum from the patients #8 and #23.

MAR patient #8, was a 76-year-old man with a history of skin melanoma. He had ring scotomas and abnormal ERGs indicating that he had MAR. The other patient, MAR #23, was a 57-year-old man with poor night vision, abnormal scotopic ERGs and abnormal color vision. He had a history of skin melanoma and thyroid cancer. There was no other clinical information available on these two patients because these sera were obtained from other institutes several years before without detailed clinical information.

## Discussion

PR, including MAR and CAR, presents visual disorders associated with systemic cancer. Antibodies against retinal cells and proteins have been detected in the sera of patients with PR suggesting an autoimmune basis for the etiology of the PR. The autoantibodies identified so far include rhodopsin, retinal transducin alpha and beta, recoverin, S-arrestin, α-enolase, carbonic anhydrase II, and heat shock protein-60 which reside abundantly in photoreceptors [1]–[10], [16]. MAR and CAR can cause bipolar cell dysfunction [7]–[12]. The results of the ERG [8], [9] and immunohistochemistry [7] studies suggested that the main target of MAR are retinal ON bipolar cells in both the rod and cone pathways. However, autoantibodies specifically reacting with a bipolar cell antigen had not been identified in the sera of patients with PR, including those with CAR and MAR. In the current study, we identified autoantibodies against TRPM1, a component of the ON bipolar cell transduction channel negatively regulated by Goα in the mGluR6 signaling pathway [13]–[15], in the sera of one CAR patient and two MAR patients. The CAR patient exhibited a dysfunction of ON bipolar cells, and to our knowledge, this is the first report on an autoantibody against a bipolar cell antigen in the serum of PR patients affecting the ON bipolar cell function.

Previously, we isolated a mouse *TRPM1-L* cDNA corresponding to the human long form of *TRPM1*, and found that the TRPM1-L protein is developmentally localized at the tips of the ON bipolar dendrites co-localizing with mGluR6, but not on OFF bipolar cells [13], [14]. The *TRPM1* null mutant mouse completely loses the ON bipolar cell photoresponses to light, indicating that TRPM1 plays a critical role in the synaptic transmission from photoreceptors to ON-bipolar cells [13], [15]. In addition, we demonstrated using a CHO cell reconstitution system that TRPM1-L is a nonselective cation channel which is negatively regulated by Goα downstream of the mGluR6 signaling cascade in ON bipolar cells [13]. Recently, four groups including ours independently reported that mutations of human *TRPM1* are associated with the complete-type of congenital stationary night blindness (cCSNB), an inherited human retinal disease [17]–[20]. cCSNB is a non-progressive retinal disease characterized by congenital night blindness with a moderate decrease in visual acuity and myopia [21]-[24]. Previous ERG studies have suggested that the defect in cCSNB patients lies in the signal transmission from photoreceptors to ON bipolar cells in both the rod and cone pathways [25]–[28]. We have identified five different mutations in our three cCSNB patients, and have shown that these mutations lead to either abnormal TRPM1 protein production or mislocalization of the TRPM1 protein in bipolar cell dendrites [17]. These results suggest that TRPM1 plays a critical role in mediating the photoresponses of ON bipolar cells in humans as well. Based on these findings, we hypothesize that the ectopic expression of TRPM1 in tumor cells of some CAR and MAR patients may result in aberrant production of autoantibodies to TRPM1 through B-lymphocytic responses [29]–[32]. These antibodies may react to the TRPM1 protein in retinal ON bipolar cells resulting in dysfunction of the TRPM1 transduction cation channel downstream of the mGluR6 signaling cascade. However, we could not confirm whether TRPM1 is expressed in the tumor cells of the three PR patients examined in this study [29] because tumor samples were not available.

Another question regarding the disease mechanism underlying PR is whether the binding of TRPM1 autoantibody to bipolar cells results in the cell death or dysfunction of bipolar cells. As far as we examined the retinal structure of the CAR patient using a spectral domain optical coherence tomography (SD-OCT) retinal imaging device, the structure of the retinal bipolar cell layer appeared to be well preserved even three months after the onset of symptoms (Fig.1D). This suggests that the autoantibodies reacting to TRPM1 cause dysfunction of the ON bipolar transduction pathway rather than bipolar cell death. However, further studies are needed to clarify the exact disease mechanism.

In the sera of MAR patients, several types of autoantibodies against retinal proteins have been reported, including the 22 kDa neuronal antigen GNB1, rhodopsin, S-arrestin, and aldolase-A and -C [10], [16], [33], [34]. We initially considered that TRPM1 might be a major MAR target antigen, because TRPM1 is exclusively expressed in retinal ON bipolar cells. However, autoantibodies against TRPM1 were detected in only two out of 26 MAR. patients' sera (7.7%, Fig. 4A, B). We tested whether the sera of one CAR patient and 26 MAR patients recognized human mGluR6, which is specifically expressed in ON bipolar cells, however, none of the sera exhibited a significant band in Western blot analysis (data not shown). Thus, antigens other than TRPM1 or mGluR6 may be involved in the pathogenesis of a large proportion of MAR.

Immunohistochemical analyses using the serum of the CAR patient showed labeling in the inner nuclear layer and outer plexiform layer of the adult rhesus monkey retina (Fig. 3D–G), where the bipolar cell bodies and dendrites reside, respectively. This immunostaining pattern is somewhat similar to our previous immunostaining results on the mouse retina with specific antibody against mouse TRPM1-L, which corresponds to the human TRPM1 long form [13]. Other labeling was also observed in the amacrine cells and ganglion cells. The reason for the immunoreactivity with these cells is uncertain, however, it may be due to the presence of other autoantibodies against amacrine cell and ganglion cell antigens. Lu *et al.* reported the presence of various different autoantibodies in the serum of a single PR patient [10]. If this is the case, it may explain why our CAR patient displayed severely reduced visual sensitivities in the visual field tests (Fig. 1A) unlike cCSNB patients with TRPM1 mutations[17]. It should be noted that we did not confirm whether there are any antoantibodies against TRPM1 in the sera of normal subjects by using a large number of samples. However, this possibility is thought to be low, because Shimazaki *et al.* reported that the molecular weights of the IgGs with observed anti-retinal reactivity in 92 normal sera were smaller than 148 kDa, which is smaller than the TRPM1 molecular weight of ∼200 kDa, although relatively high molecular weight reactivity was not intensively investigated [35].

One limitation of the current study is that we could not obtain detailed information on the two MAR patients, MAR #8 and #23, associated with the TRPM1 autoantibody. We confirmed that these two patients had skin melanomas accompanying the visual disturbances, but could not obtain a more detailed clinical history or data on visual acuity, visual field, or ERGs because these sera were sent from different hospitals several years ago. Thus, we do not know whether these two MAR patients really had retinal ON bipolar cell dysfunction. Further prospective studies of the TRPM1 autoantibodies in large numbers of MAR patients are needed.

In conclusion, our study suggests that TRPM1 may be one of the causative antigens responsible for PR associated with ON bipolar cell dysfunction.

### Note added in proof

During the course of revision process of this manuscript, Dhingra *et al. (*J. Neurosci. 31, 3962–3967, 2011) independently reported the presence of autoantibodies against TRPM1 in two MAR patients. Our study reports on autoantibodies against TRPM1 in CAR serum in addition to MAR sera.

## Materials and Methods

### Subjects

The Nagoya University Hospital Ethics Review Board approved this study (approval ID 1131). Of the PR patients that were examined in the Nagoya University Hospital, one PR patient with lung cancer and ON bipolar cell dysfunction was studied in detail. The examinations included routine ophthalmological and electrophysiological tests. In addition, immunohistochemical and Western blot analyses were performed using the serum of this patient. The procedures used conformed to the tenets of the Declaration of Helsinki of the World Medical Association. A written informed consent was obtained from the patient after he was provided with sufficient information on the procedures to be used.

We also obtained sera of 26 patients with MAR from two hospitals in Japan (Chiba University Hospital and Iwate Medical University Hospital) and Ocular Immunology Laboratory in the USA (Casey Eye Institute) for Western blot analysis.

### Ophthalmologic examinations

The ophthalmologic examination included best-corrected visual acuity, biomicroscopy, ophthalmoscopy, fundus photography, fluorescein angiography, static perimetry, and spectral-domain optical coherence tomography (SD-OCT). Static visual fields were obtained with the Humphrey 30-2 program (Carl Zeiss, Dublin, USA), and the results are shown in gray scale. SD-OCT was performed with a 9-mm horizontal scan through the midline with 50 averages (Spectralis HRA+OCT; Heidelberg Engineering, Vista, CA).

### Electroretinograms (ERG)

Full-field ERGs were elicited with a Ganzfeld dome and recorded with a Burian-Allen bipolar contact lens electrode. The ground electrode was attached to the ipsilateral ear.

After 30 minutes of dark-adaptation, a rod response was elicited with a blue light at an intensity of 5.2×10^−3^ cd-s/m^2^. A cone-rod mixed maximum response was elicited by a white flash at an intensity of 44.2 cd-s/m^2^. A cone response and a 30 Hz flicker response were elicited by a white stimulus of 4 cd-s/m^2^ and 0.9 cd-s/m^2^, respectively, on a blue background of 30 cd/m^2^. Full-field cone ERGs were also elicited by long-duration flashes of 100 ms using a densely packed array of white LEDs. The array was positioned at the top of the Ganzfeld dome and covered by a diffuser. The stimulus intensity and background illumination measured in the dome was 200 cd/m^2^ and 30 cd/m^2^, respectively. Responses were amplified by 10K and the band pass was set to 0.3 to 1000 Hz. The data were digitized at 4.3 kHz, and 5 to 20 responses were averaged (Neuropack, Nihonkohden, Tokyo, Japan).

### Immunohistochemistry

For immunohistochemistry, patient and normal sera (300 µl) were purified using the Melon Gel IgG purification kit according to the manufacturer's protocol (Pierce Biotechnology, Rockford, IL) to remove IgM, and purified sera were concentrated by Amicon Ultra 100 (Millipore, MA). The rhesus monkey eye cup was fixed with 4% paraformaldehyde in PBS for 30 min at 4°C. The samples were cryoprotected with 30% sucrose in PBS and embedded in OCT compound (Sakura Finetechnical, Tokyo, Japan). These tissues were sliced with a Microm HM 560 cryostat microtome (Microm Laborgeräte GmbH, Walldorf, Germany) into 14 µm. Sections were washed twice in PBS for 5 min, permeabilized with 0.1% Triton-X100/PBS, then washed with PBS 3 times for 5 min, and incubated with PBS containing 4% donkey serum for 1 hr to block samples. For the immunoreaction, the samples were incubated with a purified normal or CAR serum (1∶300) diluted in blocking buffer at 4°C overnight. After PBS-washing, these samples were incubated with a DyLight-488 conjugated donkey anti-human IgG (H+L) (1∶400) as a secondary antibody (Jackson Immunoresearch Laboratories) at room temperature for 1 hr and washed with PBS.

### Transfection and Western blot analyses

HEK293T cells were cultured in D-MEM containing 10% fetal bovine serum (FBS; Nissui, Tokyo, Japan). These cells were grown under 5% carbon dioxide at 37°C. The calcium phosphate method was used to transfect the cells. Transfected cells were incubated at 37°C for 48 hrs, and then harvested for further analysis. The proteins extracted from the cells were separated by SDS–PAGE on a 7.5% precast gel (ATTO, Tokyo, Japan), and then transferred to a polyvinylidene difluoride membrane using the Invitrogen iBlot system (Invitrogen, Carlsbad, CA, USA). The membrane was incubated with primary antibodies, mouse anti-Flag (1∶1,000; Sigma, St Louis, MO), sera from patients (1∶100), normal human serum (1∶100), or mouse anti-β-actin (1∶5,000; Sigma). The membrane was then incubated with a horseradish peroxidase-conjugated goat anti-mouse IgG (1∶10,000; Zymed Laboratories, San Francisco, CA) or donkey anti-human IgG (1∶10,000; Jackson Immuno Research Laboratories, West Grove, PA) as secondary antibodies. The bands were developed using Chemi-Lumi One L (Nacalai Tesque, Kyoto, Japan).
